# Multi-Scale Hybrid Network for Polyp Detection in Wireless Capsule Endoscopy and Colonoscopy Images

**DOI:** 10.3390/diagnostics12082030

**Published:** 2022-08-22

**Authors:** Meryem Souaidi, Mohamed El Ansari

**Affiliations:** 1 LABSIV, Computer Science, Faculty of Sciences, University Ibn Zohr, Agadir 80000, Morocco; 2 Informatics and Applications Laboratory, Computer Science Department, Faculty of Sciences, University of Moulay Ismail, Meknès 50070, Morocco

**Keywords:** deep transfer learning, multi-scale encoding, weighted feature maps fusion, image augmentation, polyp, inception module, single-shot multibox detector (SSD), wireless capsule endoscopy images (WCE)

## Abstract

The trade-off between speed and precision is a key step in the detection of small polyps in wireless capsule endoscopy (WCE) images. In this paper, we propose a hybrid network of an inception v4 architecture-based single-shot multibox detector (Hyb-SSDNet) to detect small polyp regions in both WCE and colonoscopy frames. Medical privacy concerns are considered the main barriers to WCE image acquisition. To satisfy the object detection requirements, we enlarged the training datasets and investigated deep transfer learning techniques. The Hyb-SSDNet framework adopts inception blocks to alleviate the inherent limitations of the convolution operation to incorporate contextual features and semantic information into deep networks. It consists of four main components: (a) multi-scale encoding of small polyp regions, (b) using the inception v4 backbone to enhance more contextual features in shallow and middle layers, and (c) concatenating weighted features of mid-level feature maps, giving them more importance to highly extract semantic information. Then, the feature map fusion is delivered to the next layer, followed by some downsampling blocks to generate new pyramidal layers. Finally, the feature maps are fed to multibox detectors, consistent with the SSD process-based VGG16 network. The Hyb-SSDNet achieved a 93.29% mean average precision (mAP) and a testing speed of 44.5 FPS on the WCE dataset. This work proves that deep learning has the potential to develop future research in polyp detection and classification tasks.

## 1. Introduction

Gastrointestinal cancer is the third major cause of death worldwide [1]. Colorectal cancer begins as a benign growth of glandular tissue in the colonic mucosa, known as adenomatous polyps, which may turn into malignant tumors over time. The number of patients affected by this disease has increased considerably in recent years [2,3,4]. The non-invasive technique of wireless capsule endoscopy (WCE) is widely utilized in clinics to examine the GI tract as an advanced medical imaging technology [5]. Contrary to traditional colonoscopy devices, it enables physicians to fully visualize the inner cavities of the small bowel from the inside without pain or sedation [6,7]. However, many circumstances hinder the diagnosis process; some polyps may be overlooked due to their small size, low illumination inside the GI tract, or the skills of the gastroenterologist. A WCE produces two or more color images per second, which lasts 8 hours, to capture an approximation of 55,000 frames per patient. A large amount of data makes the task of detection laborious and tedious for trained endoscopists to identify suspicious areas and manually locate polyp regions in each WCE frame. Therefore, a computer-aided system (CAD) is required, which may help clinicians automatically locate large and small polyps and reduce the miss rate. Even for highly-skilled clinical practitioners, the detection process is more difficult owing to the complicated characteristics of the polyp regions (shape, texture, size, and morphology). Recently, many polyp detection solutions have been proposed to assist endoscopists in providing an automated system acquiring some knowledge without requiring the physical presence of the specialists [8,9]. Deep learning (DL) architectures have rapidly grown in the medical image analysis field, owing to their superior performance in image classification compared to the handcrafted methods [10,11,12,13,14]. This study aimed to classify abnormalities and automatically detect and localize polyp regions on both colonoscopy and capsule endoscopic images [15,16,17,18]. The transfer of information between the CNN blocks can automatically learn features from raw data and avoid the obstacles of manual feature extraction. In WCE polyp detection, the lack of public and annotated datasets pushed most previous studies to utilize private data sets. Other studies on colonoscopy employed public data sets according to their initiatives (e.g., MICCAI 2015 sub-challenge on automatic polyp detection in the colonoscopy). Region-based object detection and scale variations are the primary focuses of computer vision. In this context, CNN-based object detectors can be divided into two categories: two-stage detectors, such as R-CNNs [19], and their many variants (Faster R-CNN [20], R-FCN [21], etc.), and one-stage detectors (YOLO [22], SSD [23], etc.). Two-stage detectors show low speed and high computation rates by selecting a one-scale feature map and using a fixed receptive field, which limits the practical application of deep learning in small polyp region detection. Thus, two-stage detectors are accurate but relatively slow compared to one-stage detectors that map image pixels directly to the coordinates of the bounding boxes. One-stage detectors are more accurate for speed and memory but sacrifice precision for small object detection. Recently, many researchers have effectuated corresponding research on improving the small object detection ability of SSD models. However, the trade-off between accuracy and speed is still the goal of object detection research in medical image analysis. The SSD model’s detector succeeded in preserving the location information in either shallower or deep layer networks. However, semantic information may be lacking in feature maps generated by shallow layers, particularly for small polyp regions, resulting in performance degradation over time.

The motivation of this study is to achieve an encouraging mean average precision (mAP) for polyp detection purposes while reducing the running time by implementing tremendous deep learning frameworks. For that reason, we redesigned a new SSD model using the inception-v4 (as a backbone), which can solve the problem of over-fitting and increased computation during the optimization process. Thus, the inception frameworks can capture more target information compared to the VGG networks without increasing network complexity. The inception-v4 has a more uniform simplified architecture and more inception modules than inception-v3. However, it may not be necessary to add more inception layers to obtain significant increases in the performances as stated in [24]. Therefore, a weighted mid-fusion of two successive modified inception-A modules of inception v4 is introduced in order to replace the conv4_3 layer of the VGG16 network for further contextual and semantic information extraction. The modified version of inception v4 aims to reduce the number of layers used to speed up the run time with remarkable gain in precision.

Inspired by the feature fusion single-shot multibox detector (FSSD) [25] and the GoogLeNet inception v4 [26] Szegedy et al. 2017), this paper proposes a hybrid network-based single-shot multibox detector (Hyb-SSDNet) to tackle the problem of small polyp region detection in both WCE and colonoscopy frames. To achieve this goal, the proposed method benefits from the powerful architectural designs of inception modules to produce ultimate architecture used as the backbone of the SSD detector. Indeed, it replaces the classical strategy of alternating convolutional and pooling layers of the shallow part of the VGG16 network with stacked inception modules. Since inception networks tend to be very deep, it is natural to effectively redesign them in the higher part of inception v4 (backbone) consistent with the VGG16 architecture’s small polyp target detection. This would allow the Hyb-SSDNet framework to reap all the benefits of inception blocks while retaining its computational efficiency; thus, enabling the network to detect polyp patterns of various sizes within the same layer and avoid heavy parameter redundancies. To give higher importance to the region of interest, we designed a weighted mid-fusion module (WFM) that enhances more contextual features in shallow layers by assigning weights to different channels and positions on the feature maps. The simplest strategy of concatenating mid-level inception modules is aimed at incorporating contextual and semantic information into deep networks and constructing a multi-scale feature map. Finally, a downsampling block was applied to generate a new feature pyramid that is fed to the multibox detectors to produce the final detection results consistent with the SSD process. Using proper fine-tuning parameters, we conducted extensive experiments on the WCE and the challenging CVC-ClinicDB and ETIS-Larib datasets. The results show that the Hyb-SSDNet obtains a higher mAP than the conventional SSD with gains of 16.09, 17.43, and 16.98 points on the WCE, CVC-ClinicDB, and ETIS-Larib datasets, respectively, especially for small polyp regions with small speed drops.

The main contributions of this paper are as follows:

1. The application of a lightweight version of inception v4 architecture as the backbone to improve the SSD detector ability in small polyp detection.
2. A weighted mid-fusion block of two adjacent modules of the modified version of inception-A is used to replace the Conv4_3 layer of the VGG16 network; thus, tackling the problem of missing target details.
3. The filter numbers of the first convolution layers in the stem part of the inception v4 backbone were modified from (32 to 64) to capture more patterns and relevant information similar to the original SSD.
4. The modification of the inception v4 model through the reduction of layers is used to achieve faster speed while maintaining the computational cost.
5. The Hyb-SSDNet uses a weighted mid-fusion block to add new convolution layers to construct the multi-scale feature pyramid differently from the conventional SSD.
6. The Hyb-SSDNet model robustness is verified through repeated experimentation on three well-known datasets in the field (WCE, CVC-ClinicDB, and ETIS-Larib) for a fair comparison with the competitor’s state-of-the-art methods.
7. The discussion of the advantages and limitations of the proposed framework.

The remainder of this paper is organized as follows. Section 2 presents the related studies. Section 3 describes the proposed Hyb-SSDNet model in detail. Section 4 reports the experimental results and compares them with those of other models. Finally, the conclusions are presented in Section 5.

## 2. Literature Review

### 2.1. Hand-Crafted and CNN Methods

Automatically detecting polyps in WCE images is a challenging task because of their variations in terms of size, shape, and morphology. Recently, significant progress has been made in investigating handcrafted features for gastrointestinal classification purposes. In this regard, a framework-based pyramid histogram for polyp classification based on T-CWT and gamma parameters were presented in a previous study [6]. Understanding these biological mechanisms remains highly complex. Thus, handcrafted features encode only some parts and neglect the intrinsic information of the entire frame. Recently, with the rapid development of CNN, several attempts have been made to classify colonoscopy polyp abnormalities using existing deep learning frameworks, such as VGGNet [27], GoogLeNet [28], and ResNet [29]. Limited by the size of medical datasets owing to privacy concerns, a proper fine-tuning setting leads to good results in many fields compared to retraining architectures from scratch, especially in the context of colonoscopy/endoscopy polyp recognition [30]. This is the main motivation behind transferring knowledge to wireless capsule endoscopy polyp detection tasks.

### 2.2. SSD-Based and Other Object Detectors

Polyps localization in WCE/colonoscopy images by drawing a bounding box around the localized region is the purpose of this paper, whereas polyp classification is conducted directly as an internal stage of localization. The frameworks of domain-specific object detection methods are primarily categorized into two types. One is the two-stage algorithm that first generates region proposals and then classifies each proposal into different object categories (e.g., Faster R-CNN [31]). The second one is a one-stage algorithm based on regression (e.g., YOLO [32] and SSD [33]). The authors of [34] proposed an improved version of the mask R-CNN framework for polyp detection and segmentation tasks. Jia et al. [35] presented a two-stage framework based on deep learning for automatic polyp recognition in colonoscopy images. An improved version of the CNN algorithm was proposed by TASHK et al. [36], which used DRLSE to automatically locate polyps in an image. However, a meta-analysis showed that the detection speed of the two-stage algorithm is slow, making it difficult to meet the real-time requirements of polyp detection even if it performs high localization and object recognition performance. In contrast, the one-stage algorithm achieved a high inference speed by using the predicted boxes from the input images directly, without the region proposal step. Misawa et al. [37] proposed a polyp detection system based on YOLOv3, which achieved real-time detection with over 90% sensitivity and specificity. The advantages are that there is only one processing step, so a preliminary step to extract an ROI is not performed. However, the limitation of the YOLO algorithm is that it struggles with small objects within the image due to the spatial constraints of the algorithm, especially for small polyp detection purposes. Liu et al. [38] proposed a deep framework-based single-shot detector (SSD) that uses ResNet50 and VGG16 architectures as backbone networks to detect polyps in colonoscopy videos. However, the traditional SSD-based VGG16 network only uses a 1×1 and 3×3 convolution kernel for one convolution layer making its feature extraction ability inefficient. Therefore, a more powerful backbone (such as inception v4) is needed to strengthen the polyp detection process. Some attempts have been made to address the problems of small polyp detection using the SSD algorithm. Zhang et al. [39] presented an architecture (SSD-GPNet) based on the SSD model in which they combined low-level feature maps with the deconvolution of high-level feature maps. Jeong et al. [40] proposed an enhanced version of the SSD model, in which they fully utilized the direction information of the feature maps by performing a simple concatenation and deconvolution operation. These improvements made the Rainbow SSD suitable for small-target detection. Although some attempts have been made to improve the accuracy of small polyp region detection by simplifying the SSD architecture, the addition of deconvolution layers leads to excessive computational complexity at the expense of speed. Within the same scope, Sheping et al. [41] proposed an enhanced SSD network-based dense convolutional network (DenseNet), in which a residual block was established before object prediction to further improve the model performance. They performed a fusion mechanism of multi-scale feature layers to enhance the relationships between the levels in the feature pyramid. However, information from multiple scales was treated equally during fusion. For feature extraction enhancement, a weighted mid-fusion to capture more informative patterns and contextual information is needed, giving weight to more important features and reducing the model complexity while maintaining high execution speed; thus, reaching the requirements for a complete detection process. The ability to perform both real-time detection and high precision is an indispensable factor in clinical applications [42]. A lightweight concatenation of mid-level visual and semantic features is investigated to fully utilize the synthetic information that leads to the improvement in the performance of small polyp detection in WCE images.

### 2.3. Single-Shot Multibox Detector (SSD)

**Figure 1:**
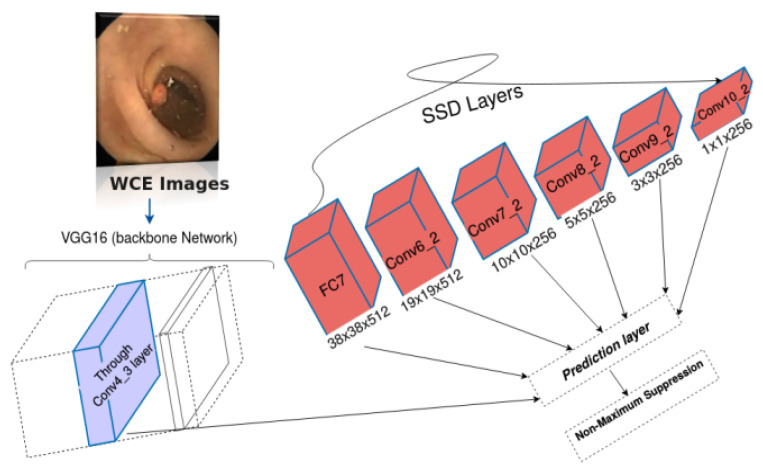
Framework of the traditional SSD.

The single-shot multibox detector (SSD) uses VGG16 as the backbone network and is truncated with other convolutional layers [23]. SSD uses ConvNet to generate a series of fixed-size bounding boxes and scores. Subsequently, it employs a non-maximum suppression (NMS) method for final detection. Figure 1 shows the SSD300 design framework separated into two parts; backbone and pyramid networks. The input sizes of the original images are 300 × 300 × 3. The VGG-16 network is used in the SSD300 model as the backbone, the first part constitutes four layers, and the second part is the application of a convolution operation to the Conv4_3 layer of the VGG16 network to generate five additional layers [18]. Shallower layers are investigated to predict smaller regions, while deeper layers for bigger ones. However, the problem of small object detection persists as the semantic information is not well extracted in shallower layers.

### 2.4. Feature Pyramids Hierarchy

For object detection in medical imaging, various deep network architectures have been investigated to trade-off between precision and speed. Faster R-CNN [31] and R-FCN [21] perform one-scale feature maps to propose anchors for different scales. However, they cannot efficiently detect multi-scale objects as well as regions of small sizes. FPN [43] and DSSD [44] have used bottom-up and top-down architectures, respectively. For the rapid detection process, layer-by-layer feature map fusion is not recommended. The traditional SSD [23] uses the features of shallower layers, concatenates, and scales them from bottom to top to make predictions. Then, it generates a pyramidal feature map. Inspired by the feature fusion module, the proposal adopts the FSSD network structure [25]. However, the feature pyramid network has been altered to enhance the fusion effect in which an improved inception network is proposed to optimize SSD for improving the small object feature extraction ability in the shallow network and to tackle the problem of scale variations in detecting small polyp regions more effectively. The detailed information is presented in the following sections.

## 3. Materials and Methods

**Figure 2:**
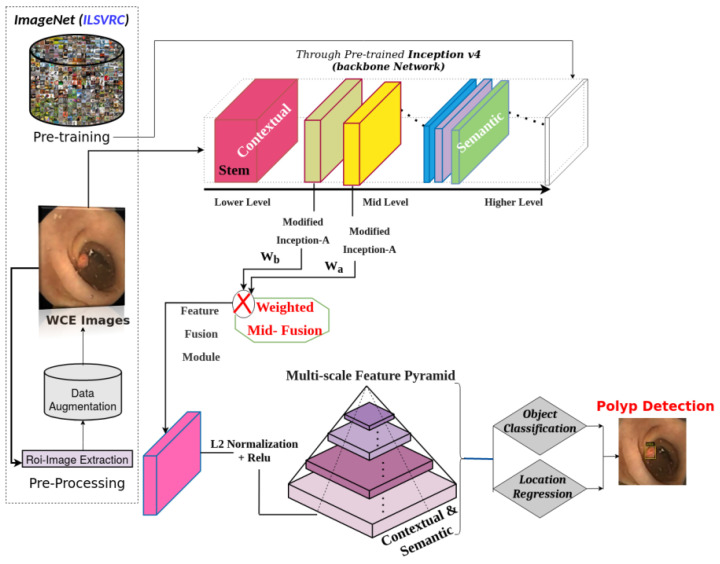
Flowchart of the proposed Hyb-SSDNet method for small polyp detection in WCE images.

A systemic overview of the Hyb-SSDNet architecture for polyp detection is shown in Figure 2. The low-level feature layer with rich object information is not well reused in the conventional SSD using the VGG16 backbone, and the polyp region information is partially lost in the multi-layer transmission process. Therefore, the resulted feature information at each layer is unbalanced. To boost the detection effect of the model, an improved version of the SSD model-based inception v4 network (as the backbone) is proposed with further modification in regards to the deep network part. This study aimed to develop an optimization method for the detection results without sacrificing speed by fully utilizing the synthetic information of all the feature layers and increasing the effective receptive field. Thus, more object feature information can be extracted. We first describe the pre-processing step, in which the surrounding black regions in the WCE images are removed to extract the region of interest (ROI) patches that provide only useful information. The training phase of Hyb-SSDNet is divided into two parts: (i) data augmentation strategies to handle overfitting in deep learning models owing to the data insufficiency problem. Then, we pre-trained the Hyb-SSDNet model on the ILSVRC CLS-LOC dataset [45] and fine-tuned it on the WCE/colonoscopy polyp datasets. (ii) The backbone network inception v4 for feature extraction (including a weighted mid-fusion block of the inception modules) for small polyp detection and the classification sub-network on multi-scale feature maps.

### 3.1. Mid-Fusion Block

**Figure 3:**
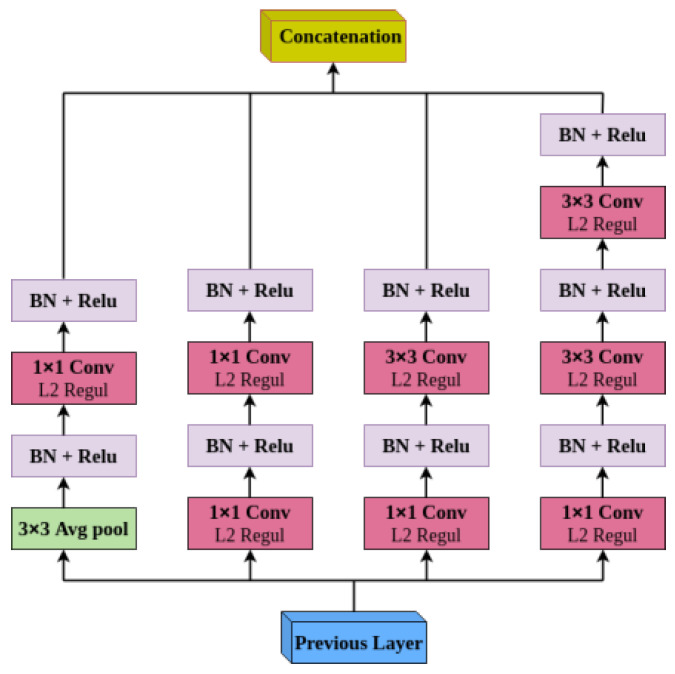
Modified inception structure.

Different multi-modal fusions have been widely used in deep learning. Mid-fusion operates independently at each scale. It then merges and passes them to complete the network processes. However, information from multiple scales is treated equally during fusion. Capturing variance in visual patterns as well as more informative patterns in class prediction is one of the requirements of the complete classification approach. Besides, the traditional SSD-based VGG16 network only uses the 1×1 and 3×3 convolution kernel for one convolution layer, making its feature extraction ability inefficient. To address this shortcoming, two weighted inception-A modules based on inception v4 were adapted to the mid-fusion block, giving more weight to important features. The modified inception-A module is presented in Figure 3. To enhance the feature extraction, the network width is increased by exploiting sparsity in the network connectivity. A cascade convolution layer composed of two convolution kernels with different sizes and an average pooling layer are combined in parallel. First, the 1 × 1 convolution reduces the dimension before the convolution operation and after the pooling operation, which significantly reduces the calculation cost. The inception-A module of (inception v4 network) uses two 3 × 3 continuous convolutions instead of the 5 × 5 convolution kernel to extract more detailed object features and improve the calculation speed. Finally, the information of different receptive fields is concatenated in one single layer in order to improve the feature representation ability of the network. The L2 weight regularization is added to the convolutional layers, the most common type used for neural networks with a sensible default weighting of 0.00004 specifying the type of regularization as He_normal initializers. In addition, after the convolution layer, batch normalization (BN) and ReLU activation functions are used to accelerate the network training speed and enhance the detection robustness.

**Figure 4:**
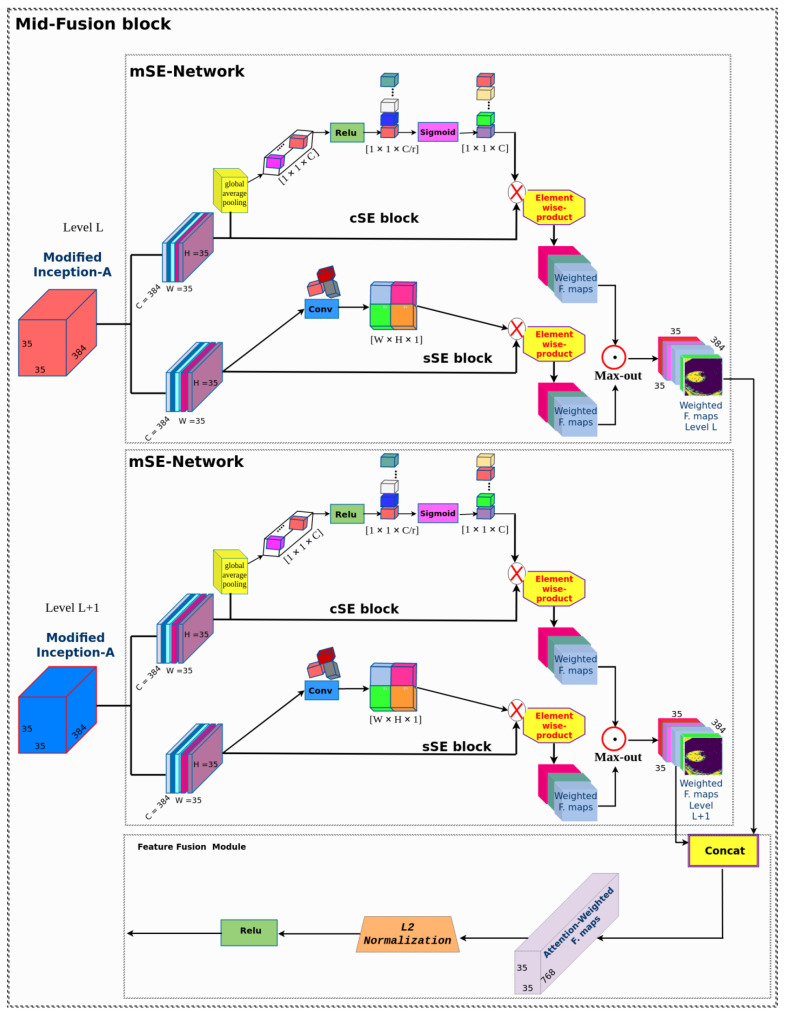
The mid-fusion framework. Input feature maps were created at two successive inception modules of the inception v4 network to encode contextual information. All scale sizes are 35 × 35 × 384. Extracted features were passed to the mSE-Network to generate the score maps, reflecting the importance of features at different positions and scales. Weighted features were then concatenated and normalized to complete the network process.

The main fusion structure is shown in Figure 4. We considered $S_{L_{i}}$ as the mid-level inception module feature and $S_{L_{i+1}}$ as its adjacent inception module feature maps. After extracting mid-level features at two scales, we apply the mSE-Network to each inception feature map in each channel (F) and spatial position, enabling the extraction of the most representative features in highlighting the region of interest computed as:

(1) $${\bar{S}}_{L_{i}}^{F}=\left(mSE-Network\left(S_{L_{i}}\right);mSE-Network\left(S_{L_{i+1}}\right)\right)$$

The ${\bar{S}}_{L_{i}}^{F}$ and ${\bar{S}}_{L_{i+1}}^{F}$ have the same channel sizes and are fused using a simple concatenation strategy. In the literature, there are other fusion approaches, such as element-wise summation, element-wise multiplication, averaging process, etc. To verify the best concatenation strategy, several experiments were conducted. Inspired by the [46] connection module, a simple concatenation strategy was adopted in this study for the global feature fusion module, as it is more effective. The concatenation operation provides more feature representations for feature maps of the same size. The map merging operation can be described as:

(2) $$S_{HL}^{{}^{\prime }}=D\left({\bar{S}}_{L_{i}}^{F};{\bar{S}}_{L_{i+1}}^{F}\right)$$

where *D* is the feature concatenation operation, ${\bar{S}}_{L_{i}}^{F}$ and ${\bar{S}}_{L_{i+1}}^{F}$ are the channel-adjusted feature maps from two successive mid-level layers, and $S_{HL}^{{}^{\prime }}$ is the fused feature map producing a new high-resolution image that contains both rich semantic features and location information. An L2 normalization operation and ReLU activation were then applied to all filters after the concatenation procedure because the feature values in the layers were significantly different in scale. The feature fusion module also localizes small polyp regions by assigning higher weights to pixels containing the object class. Furthermore, the fusion block tends to increase the discrimination capacity of the network by highlighting small polyp edges in some of the feature maps. Thus, the most informative features of the background were extracted and discriminated from the object in the weighted feature maps. In this study, the mid-fusion block is used to generate spatial and multi-scale score maps for the features extracted from two successive inception modules at the mid-level of the inception v4 network. Polyp regions show great variation in shape, size, and orientation at different scales. This is the main motivation behind using a heterogeneous weighted feature fusion of polyp regions. Thus, making the network sensitive to unique contextual image features improved the classification performance for polyp regions.

#### mSE-Network

This is motivated by the squeeze-and-excitation network’s success [47] in showing significant improvement in the performance related to medical image analysis. Hyb-SSDNet uses a learnable mechanism, highlighting the most salient feature maps, and avoiding no-useful information of background. The structure of the squeeze-and-excitation block (mSE-Network) used in this proposal is shown in Figure 4.

**cSE Block** The squeeze-and-excitation network uses a squeeze operation as a primary process by applying a global average pooling (Shen et al., 2015) to spatially compress the input feature maps, and reduce the dimensionality from W×H×C to 1×1×C. The squeeze operation can be described in Equation (3) as follows: We consider the input feature map $Y=[y_{1},y_{2},y_{3},\ldots ,y_{c}]$, $y_{i}\in R^{H\times W}$

(3) $$x_{l}=\frac{1}{W\times H}\sum_{j}^{W}\sum_{k}^{H}y_{l}\left(i,j\right).$$

where *x* is the vector output, $x\in R^{1\times 1\times c}$ with its lth element. The spatial squeeze operation only embeds the global spatial information in the output vector (*x*). Therefore, the channel-wise dependencies will not be well encoded. Hence, the squeeze-and-excitation network uses the excitation operation to solve the channel’s weight inefficiency. One of the more significant issues of the excitation operation is to properly learn the non-linear interaction between channels as they have to be activated simultaneously. Therefore, two fully-connected layers $F_{1}\in R^{C\times \frac{c}{r}},F_{2}\in R^{\frac{c}{r}\times C}$, and the ReLU operation δ(.) were used. The excitation operation is calculated in Equation (4) as follows:

(4) $$\bar{x}=F_{1}\left(Relu\left(F_{2}\times x\right)\right).$$

where *C* and *r* mean the number of channels and the scaling parameters, respectively. To reduce the computational complexity of the modified inception-A modules, the r parameter is set to 2. Finally, a channel-wise recalibration of the output vector was performed with the input feature maps to be scaled consistently to original sizes. We consider sig as the sigmoid activation. The final step reconstructs the spatial squeeze and channel excitation block (cSE). The merging operation expressed in Equation (5) is as follows:

(5) $$\tilde{x}=\left[sig\left({\bar{x}}_{1}\right)y_{1},sig\left({\bar{x}}_{2}\right)y_{2},sig\left({\bar{x}}_{3}\right)y_{3},\ldots ,sig\left({\bar{x}}_{C}\right)y_{C}\right].$$

**sSE block:** The channel squeeze and spatial excitation (sSE block) assume the importance of squeezing the input feature map along the channels and mutually exciting them spatially. Therefore, a convolutional layer is applied to compress the input feature map. The sigmoid function is activated to obtain spatially distributed information. Finally, the sSE block processed a concatenation operation for the feature map fusion with the input feature map, computed in Equation (6) as:

(6) $$\ddot{x}=Sig\left(W_{d},Y\right)\times Y.$$

where $Y=[y^{1,1},y^{2,2},y^{3,3},\ldots ,y^{H\times W}]$, $y^{i,j}\in R^{1\times 1\times C}$ is the input feature map and $W_{d}\in R^{1\times 1\times 1\times C}$, generating a projection tensor $d\in R^{W\times H}$, $\ddot{x}$ is the final vector for the sSE block. To mutually recalibrate feature information in both spatial and channel directions, the mSE-block performs a joint version of the spatial squeeze and channel excitation (cSE) block and channel squeeze and spatial excitation (sSE) block [48], which can be calculated in Equation (7) as follows:

(7) $$YscSE\left(i,j,C\right)=max(\tilde{x}\left(i,j,C\right),\ddot{x}\left(i,j,C\right)).$$

This is based on the experiments conducted in the work of [48]. Concatenation obtains the best results compared to other strategies that target the spatial and channel squeeze excitation SE. However, it increases the channel number that implicitly increases the model complexity. Thus, we used the max-out strategy to merge the two blocks as it obtains approximately similar results to the concatenation procedure.

### 3.2. Multi-Scale Feature Pyramid

**Figure 5:**
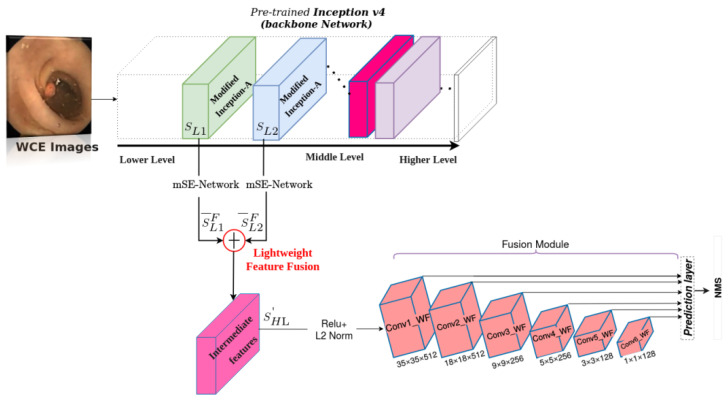
Overview of the Hyb-SSDNet architecture with a 299 × 299 × 3 input size and inception v4 as the backbone. Features from two successive modified inception-A layers ($S_{L1}$, $S_{L2}$) were fused by a mid-fusion block producing an intermediate feature representation ($S_{HL}^{{}^{\prime }}$).

**Figure 6:**
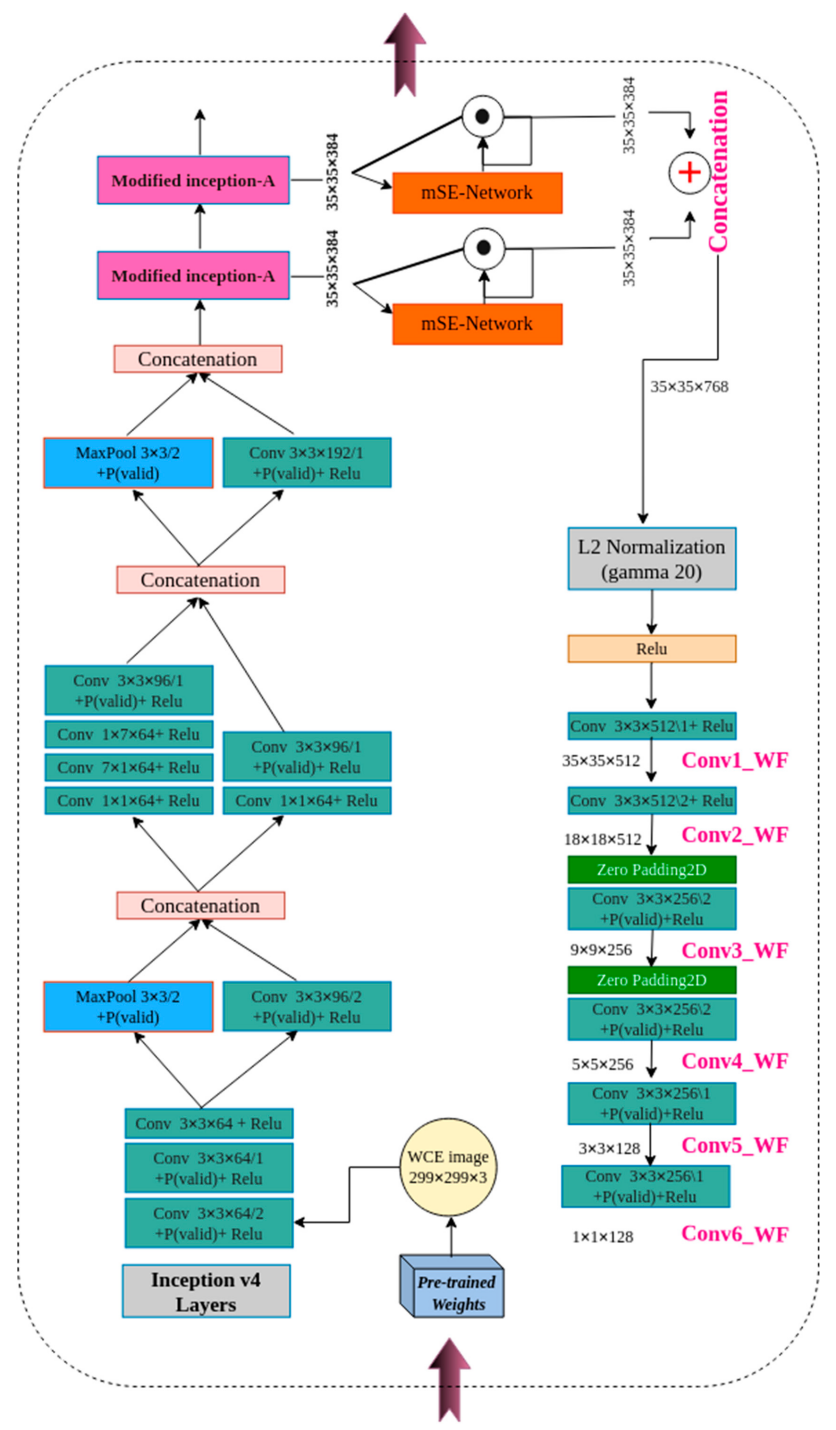
Detailed structure of the Hyb-SSDNet network.

This is motivated by FSSD [18,25]. The feature maps from the weighted feature fusion module (Figure 4) using the inception v4 network (backbone) were considered as inputs for the multi-scale feature pyramid, as shown in Figure 5. The lightweight feature fusion module ($S_{HL}^{{}^{\prime }}$) is designed to utilize a spatially coarser but a semantically richer feature map from the middle pyramid level incorporating proprieties of both high level and its relatively lower-level features. Figure 5 presents a top-down Hyb-SSDNet architecture that incorporates semantic information of shallow and mid-level layers into textural features of high levels. The entire process is as follows: First, a mid-fusion block of two adjacent inception-A modules was performed. Then, L2 normalization and ReLU activation were applied. The input size of the WCE/colonoscopy images was 299 × 299, and the network structure of the Hyb-SSDNet could be divided into two parts: the backbone network inception v4 feature extraction and feature fusion module, and multi-scale feature pyramid for polyp region detection. The filter numbers of the first convolution layers in the stem part of the inception v4 backbone were modified from 32 to 64 to reinforce the model to capture more patterns and relevant information similar to the original SSD. Sequential experiments proved that these steps enrich the semantic information of shallow and mid-level features, and improve the model performance in small polyp regions. After concatenation, a batch normalization operation was performed. The detailed structure of the model is illustrated in Figure 6. Based on experiments conducted in the work of [25], a feature map of resolution smaller than 10 px × 10 px may not contain much information to reuse. This was inspired by the feature fusion SSD (FSSD) [25]. We constructed multi-scale feature layers based on the SSD network, which added new layers, Conv6_2, Conv7_2, Conv8_2, and Conv9_2, for object classification and location regression. To ensure precision and detection speed, the Hyb-SSDNet network investigated new feature fusion module layers based on the inception v4 network, Conv1_WF, Conv2_WF, Conv3_WF, Conv4_WF, Conv5_WF, and Conv6_WF, for the detection of small polyps. The target sizes of the resulting feature layers were 35 × 35, 18 × 18, 9 × 9, 5 × 5, 3 × 3, and 1 × 1, different from those of the FSSD model.

## 4. Experiments

### 4.1. Datasets

**Figure 7:**
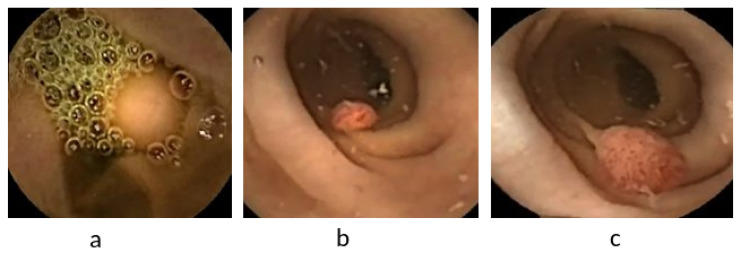
Example of WCE polyp images (**a**–**c**).

**Figure 8:**
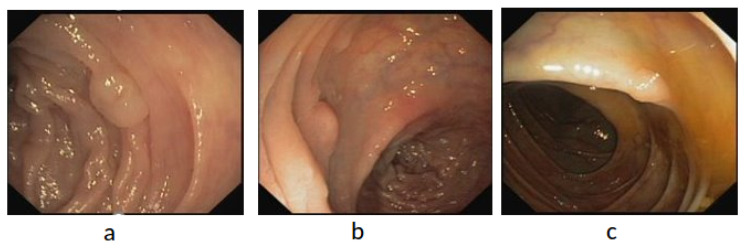
Example of CVC-ClinicDB polyp images (**a**–**c**).

**Figure 9:**
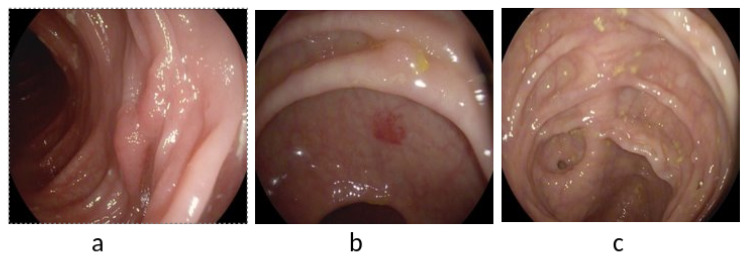
Example of ETIS-Larib polyp images (**a**–**c**).

The Fist WCE dataset was acquired from PillCam©COLON 2 polyps [49]. The original image’s resolution was 256 pixels × 256 pixels. As depicted in Figure 7, the WCE dataset contains 120 polyps and 181 normal images obtained from one patient’s VCE test. To avoid the overfitting problem owing to the small dataset size, we increased the training dataset size by the pre-processing step, as described in this section. Therefore, the revised dataset included 1250 polyp patches and 1864 normal patches. Subsequently, two trained experts reviewed and manually defined binary masks corresponding to the polyp regions covered to provide ground truths after being manually labeled and annotated as positive and negative samples. The ground truth bounding box was drawn based on the mask ground truth provided by specialists to meet the requirements of the polyp detection tools, using a graphical image annotation tool and label object bounding boxes in images (LabelImg) and corrected by experts. A popular colonoscopy dataset called CVC-ClinicDB [50] was also used in this work, which contained frames showing polyp regions of different shapes. A total of 25 colonoscopy videos were used by researchers to select at least 29 sequences containing one polyp region in every frame. Subsequently, a set of frames was selected for each sequence. As depicted in Figure 8, the CVC-ClinicDB dataset comprises 612 polyp images (size: 384 × 288; format: tiff). Experts created the ground truth by manually defining masks corresponding to the regions covered by the polyps in each frame. To meet the requirements of polyp detection tools, the ground truth bounding box was drawn based on the ground truth provided by specialists. The annotated ETIS-Larib [51] dataset was used to assess the detection results. A total of 34 colonoscopy videos were used to generate 196 polyp images of different shapes and sizes. The ground truths of the ETIS-Larib dataset were annotated by competent endoscopists members of clinical institutions as depicted in Figure 9. The colonoscopy CVC-ClinicDB [52] and ETIS-Larib [53] datasets were adopted in the 2015 MICCAI sub-challenge on automatic polyp detection. The normal and abnormal images were rescaled to 299 × 299 pixels to reach the pre-trained inception v4 network input size. We split the data into training 70%, validation 10%, and model testing 20%. The five-fold cross-validation [54] was used, in which the state and convergence of the model were checked after each epoch was completed. The hyperparameter number of iterations and learning rate were adjusted automatically during the validation data. In general, the validation set only adjusts the hyperparameters, such as the number of iterations and learning rate. Subsequently, they were adopted according to the five group performances in the models. The results were then averaged over the splits to estimate the mean average precision metric.

### 4.2. Evaluation Indexes

In this study, the mean average precision (mAP) metric was adopted to evaluate polyp detection performance. Using an intersection over union (IoU) of 0.5, the mAP is defined as the average of the average precision (AP) of all object categories. It is formulated by Equation (8).

(8) $$mAP=\frac{\sum_{q=1}^{Q}AveP\left(q\right)}{Q}$$

where *Q* is the number of queries in the set and *q* is the query for average precision.

Given a set of ground truth bounding boxes annotated by the experts, and a set of predicted bounding boxes produced by the network with a confidence score above a specific threshold (for example, 0.5), *precision*, *recall*, and *F1-score* can be defined in Equations (9) and (10) as:

(9) $$Precision=\frac{TP}{TP+FP}\;Recall=\frac{TP}{TP+FN}$$

(10) $$F-measure/F1-score=\frac{\left(2\times Recall\times Precision\right)}{Recall+Precision}$$

where *TP* denotes true positives; that is, IoU > 0.5, *FP* indicates false positives and *FN* indicates false negatives. Frame per second (FPS) is used to measure the detection speed and indicates the number of frames transmitted per second. More details regarding the evaluation metrics can be found in [18].

### 4.3. Experimental Setup

#### 4.3.1. Experimental Environment Configuration

The experiments were performed using Colab Pro Plus solution provided by Google, with a maximum RAM of 52 Gb and a disk of 166,83 Gb. All experiments were conducted using CUDA 8.0.61-1, CuDNN6.0, Keras 2.0.5, Python 3.5, h5py 2.10.0, NumPy 1.16.3, TensorFlow 1.15, TensorFlow-GPU 1.15, and OpenCV 3.1. According to the ground truth bounding boxes of the small polyp region, the same aspect ratio within a range of 1–2 was maintained for all employed datasets.

#### 4.3.2. Model Training

To verify the capability of the Hyb-SSDNet model, various indicators and parameters in the field of medical imaging were investigated and adjusted. As a primary process, the surrounding black regions containing no useful information about the WCE polyp image were removed, as they may have degraded the performance of WCE polyp detection and increased the computation time. The input images were resized to 299 × 299 × 3, and the batch size was 32. Concerning data augmentation strategies [55], we investigated flipping, rotation by $270^{\circ }$, and cropping schemes. Further details can be found in previous studies [56,57]. We pre-trained the model on the ImageNet Large Scale Visual Recognition Challenge (ILSVRC) dataset, as it showcased massive progress in large-scale object recognition over the past five years. We then fine-tuned the model on the WCE and colonoscopy datasets. This method dynamically decreases the learning rate based on the number of epochs, training times, and losses. Adaptive moment estimation (Adam) is an optimization method for the adaptive learning rate. Practically, the Adam optimizer is utilized in this work due to its efficiency with large problems. The initial learning rate is set to 0.0001, and the learning rate decay policy is approximately different from the original SSD with a drop of 0.5 and epochs_drop of 10. Other hyperparameters were adjusted, beta_1 = 0.9, epsilon of 1 × 10^−8^, and beta_2 = 0.999. Using Keras, the learning rate schedule is defined as follows: 0.0001 if epoch < 50, 0.00001 if epoch < 80, and 0.000001 otherwise. Hyb-SSDNet is an improved model for the in-depth feature fusion of SSD300. For a fair comparison with the state-of-the-art approaches, target polyp detection was used [18]. The model uses 100 epochs and 500 steps per training epoch. The mean average precision (mAP), the number of picture frames that could be detected by the model per second (FPS), and other indicators were used to evaluate the detection performance. Similar to conventional SSD [23], the same loss function was used in the Hyb-SSDNet model. The hyper-parameter neg_pos_ratio was set to 3 and the α parameter was adjusted by cross-validation to 1. More SSD_Loss details are presented in [41].

### 4.4. Results and Discussion

#### 4.4.1. Ablation Studies

In this section, we investigate the effectiveness of the different components that affected the experimental results of the main structure of the Hyb-SSDNet detector through an ablation study on the WCE, CVC-ClinicDB, and ETIS-Larib colonoscopy datasets. Different Hyb-SSDNet model settings are shown in Table 1 in which the inception v4 backbone was kept. A mid-fusion block was designed based on the middle part of the detection network to incorporate the semantic information of small polyps into the Hyb-SSDNet network. Training and testing were performed on the WCE images, or in the CVC-ClinicDB and ETIS-Larib joint training set and tested on the CVC-ClinicDB and ETIS-Larib test sets. First, we compare two mainstream normalization methods (Batch Normalization and L2 Normalization) and report their effects on the performance mAP (%) of the proposed system. As reported in Table 1 (row 5, row 7 and row10), L2 normalization yielded better results (90.11%, 93.29% and 92.53% mAP), whereas it is (88.42%, 89.96% and 89.75% mAP) using batch normalization on the WCE train and test. The CVC-ClinicDB and ETIS-Larib test sets support the results obtained on the WCE test set (col 5–row 17 and row 20) and (col 4–row 27 and row 30). Thus, it is reasonable to choose L2 normalization instead of batch normalization in mid-fusion design. Various feature fusion techniques have been investigated to determine the most appropriate one for the Hyb-SSDNet framework. Considering the WCE test set, it can be seen in Table 1 (rows 7) that the framework adopting simple concatenation to fuse and scale the feature maps of shallow and middle layers obtains the best result, 3.18 points higher than the average strategy and 4.6 points higher than element-wise summation while it costs low performances in term of mAP Table 1 (row 1 and row 8) without utilizing a weighted fusion structure. This means assigning a weighted score to each feature map in each layer and scale, giving them relative importance in contributing to small polyp detection. In addition, experiments on the CVC-ClinicDB and ETIS-Larib test set Table 1 (row 11–20) and (row 21–30) support the results obtained on the WCE images and demonstrate the high efficiency of the concatenation strategy for mid-level fusion with L2 normalization, highlighting more important features for small polyp detection.

**Figure 10:**
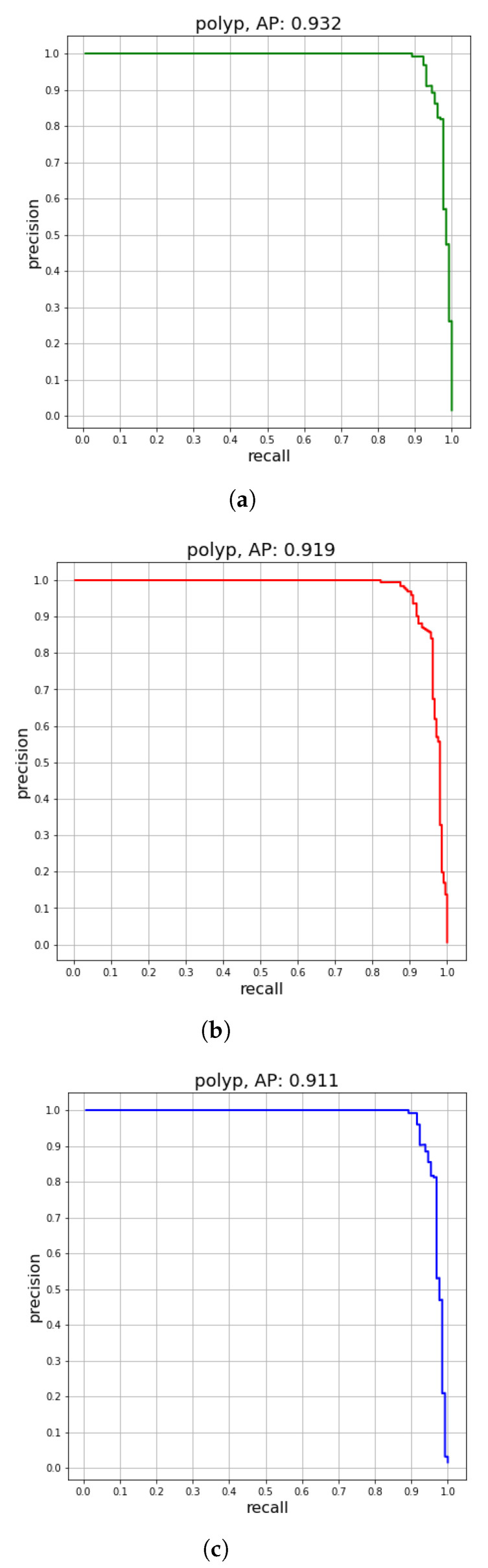
Precision vs. recall for (**a**) WCE test set, (**b**) CVC-ClinicDB test set, and (**c**) ETIS-Larib test set using the Hyb-SSDNet framework.

Table 2 lists the results of the proposed method on the WCE, CVC-ClinicDB, and ETIS-Larib test sets, as well as some state-of-the-art detector-based SSDs. The (mAP) of SSD300 using VGG16 (backbone) were 77.2%, 74.5%, and 74.12% for the WCE, CVC-ClinicDB, and ETIS-Larib test sets, respectively. The feature fusion strategy provides richer details and semantic information, showing remarkable results compared to the conventional SSD. As depicted in Table 2 (rows 5–6 and rows 15–16), FSSD300 and FSSD500 frameworks were increased by (12.58% and 9.25%), (12.76% and 9.16%), and (12.18% and 11.47%) in terms of (mAP) in WCE, CVC-ClinicDB, and ETIS-Larib test sets. By replacing the backbone network VGGNet with DenseNet-S-32-1, the DF-SSD300 model exceeded the FSSD300 by 1.46%, 2.66%, and 0.54 mAP (91.24% vs. 89.78%), (89.92% vs. 87.26%), and (86.84% vs. 86.3%) for all test sets, respectively. The detection speed decreases by half. The VGG16 network was replaced with the ResNet-101 backbone in the L_SSD model (row 8 and row 18) to perform feature fusion, which contained rich details and semantic information. The mAP of ResNet-101 was slightly better than that of FSSD300 for both WCE and colonoscopy datasets. The proposed Hyb-SSDNet algorithm outperforms the FSSD300, DF-SSD300, and L_SSD models by 3.51 points (93.29% vs. 89.78%), 2.05 points (93.29% vs. 91.24%), and 3.31 points (93.29% vs. 89.98%) on WCE dataset, while it achieves almost similar performance with the MP-FSSD network on the WCE dataset; 0.22 points (93.29% vs. 93.4%). The Hyb-SSDNet framework achieved promising results, with a small drop in speed. However, it still achieved real-time detection with 44.5 FPS, compared with DF-SSD300 and L-SSD with 11.6 FPS and 40 FPS, respectively. Figure 10a–c supports the results obtained in Table 2 as it shows the recall vs. precision plots obtained for this experiment on WCE, CVC-ClinicDB, and ETIS-Larib test sets. The Hyb-SSDNet has strong feature reuse and extraction abilities. The mid-fusion block uses a weighted concatenation of spatial features at each location and scale. Therefore, the features of unrelated regions, such as the background, are suppressed. Thus, the fusion inception modules help the model focus on real targets and improve the precision of the detection of small polyps.

#### 4.4.2. Comparison with the State-of-the-Art Method

For fair comparison experiments with the proposed Hyb-SSDNet framework, we selected the most highlighted methods in the literature-based SSDs models, YOLOv3, and DSSD, as listed in Table 3. To quantitatively compare Hyb-SSDNet with other state-of-the-art models, the performances of the trained model on the ETIS-Larib and CVC-ClinicDB training sets were evaluated on the publicly available ETIS-Larib dataset. As depicted in Table 3, the Hyb-SSDNet model obtains encouraging results of the (mAP) metric on the WCE dataset compared with other models. This is attributable to the fact that polyp abnormalities exhibit great variation in terms of color and shape. In addition, WCE, CVC-ClinicDB, and ETIS-Larib images differ in nature, texture, and illumination acquisition conditions. However, they achieved similar metrics. Additionally, the colors of the polyps vary across different images within a patient’s VCE and other patients’ examinations. Compared to previous approaches targeting polyp detection, Hyb-SSDNet achieved the best mAP, which reached 93.29%, 91.93%, and 91.10%, respectively. The joint training of the ETIS-Larib and CVC-ClinicDB datasets exceeded some competitor models or achieved approximately similar metrics on the ETIS-Larib test set. Overall, the Hyb-SSDNet shows promising results in terms of the mAP with a slight speed drop.

#### 4.4.3. Visualization of Detection Results

**Figure 11:**
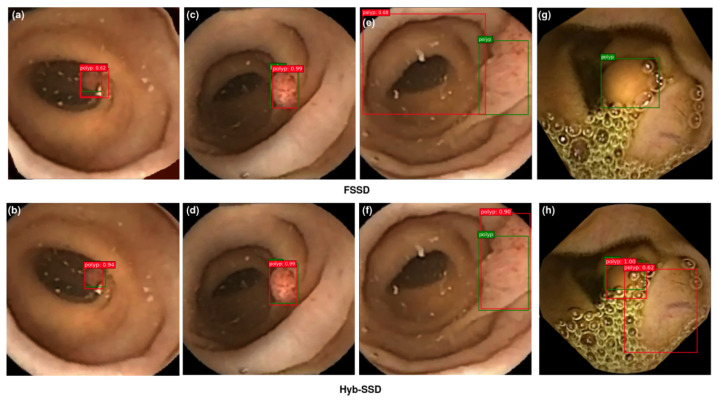
Qualitative results comparison between FSSD300 (**a**,**c**,**e**,**g**) and the proposed Hyb-SSDNet (**b**,**d**,**f**,**h**) on the WCE polyp test set. True bounding boxes with IoU of 0.5 or higher with the bounding predicted boxes are drawn in green and red colors, respectively.

**Figure 12:**
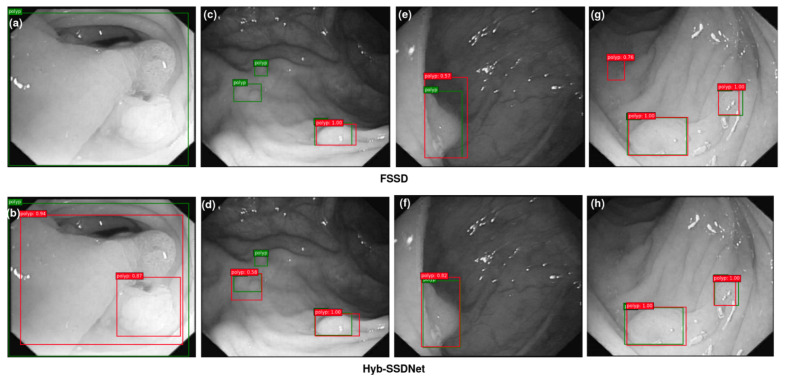
Qualitative results comparison between FSSD300 (**a**,**c**,**e**,**g**) and the proposed Hyb-SSDNet (**b**,**d**,**f**,**h**) on the CVC-ClinicDB polyp test set. True bounding boxes with IoU of 0.5 or higher with the bounding predicted boxes are drawn in green and red colors, respectively.

**Figure 13:**
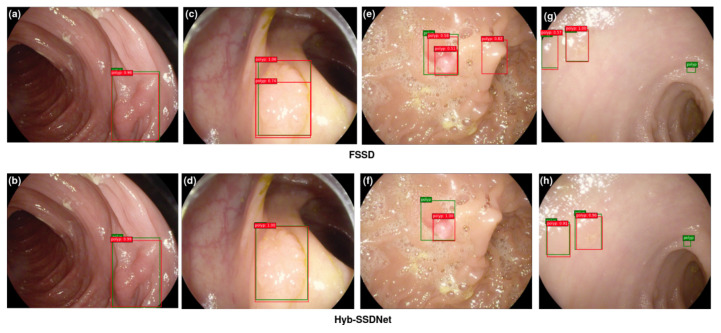
Qualitative results comparison between FSSD300 (**a**,**c**,**e**,**g**) and the proposed Hyb-SSDNet (**b**,**d**,**f**,**h**) on the ETIS-Larib polyp test set. True bounding boxes with IoU of 0.5 or higher with the bounding predicted boxes are drawn in green and red colors, respectively.

The main objective is to prove the efficiency of the proposed Hyb-SSDNet framework, not only for WCE polyp images detection but also for colonoscopy polyp localization cases (CVC-ClinicDB and ETIS-Larib). Polyp detection involves the identification of polyp regions and the rejection of parts containing normal, blurry tissues, and others showing feces or water jet sprays to clean the colon. Figure 11, Figure 12 and Figure 13 show some detection cases of the FSSD and their analogs of the Hyb-SSDNet model on the WCE, CVC-ClinicDB, and ETIS-Larib polyp test sets, respectively. The false negative rate decreased because the Hyb-SSDNet model Figure 11h and Figure 12b,d showed a relative improvement in small polyp localization from the normal intestinal mucosa in comparison with the FSSD model Figure 11g and Figure 12a,c. Specifically, the FSSD model failed to observe the context information of the object because it uses a small receptive field that can only detect smaller objects from shallow layers. Other circumstances have hindered the identification of small polyp regions. Their similarity in appearance to the surrounding normal mucosa, their complicated characteristics (color, texture, contrast, and size), and the presence of air bubbles, food, and other debris, limits the visualization of the actual extent of the lumen by capsule endoscopy. Therefore, Hyb-SSDNet performs a weighted mid-fusion module of middle layers and embeds it into the final fusion block, which can capture scene contexts and differentiate polyp edge regions from normal mucosa. Figure 11e, Figure 12g, and Figure 13c,e show the false positive identifications of the FSSD model in the normal mucosa areas of the WCE, CVC-ClinicDB, and ETIS-Larib test sets. Compared to the Hyb-SSDNet framework, Figure 11f, Figure 12h, and Figure 13d,f show some true-positive identification cases of Hyb-SSDNet in different normal mucosa areas. Regarding the WCE images, the difference in appearance between the polyp regions and the normal mucosa was not obvious. Thus, the WCE frames captured in the case of insufficient light produced poor pixels, which hindered the detection process as depicted in Figure 11h. However, the proposed Hyb-SSDNet algorithm also shows a remarkable improvement in large polyp detection for the WCE, CVC-ClinicDB, and ETIS-Larib test sets, and accurately distinguishes them from the surrounding normal areas. Limited by different debris, Figure 12b and Figure 13h show hard cases of small polyps behind the folds of the CVC-ClinicDB and ETIS-Larib test sets, respectively. Even specialists sometimes mistakenly recognize these parts when they are hidden from view behind a fold or wrapped around a fold in a clamshell fashion and find them difficult to ’resect’.

However, the proposed Hyb-SSDNet model successfully detected a hard polyp case in which the ground truth was not annotated by experts Figure 12b.

## 5. Conclusions

In this paper, an improved FSSD detection model (Hyb-SSDNet) was proposed for accurate polyp abnormality localization and detection in both WCE and colonoscopy datasets. In addition to the problem of medical ethics, researchers have been pushed to use their datasets for polyp detection owing to the lack of standard and public WCE datasets. Therefore, research in this field is limited and the performance metrics may be subjective. The lightweight version of the Hyb-SSDNet aims to model the visual appearance of small polyp regions via a weighted mid-fusion of inception modules for a polyp detection network using the inception v4 backbone. The mid-fusion block uses the modified inception-A modules to magnify the mid-level feature maps instead of using simple convolution layers. Therefore, the Hyb-SSDNet model shows promising results in the (mAP) metric with little speed drop. An efficient feature fusion module using a simple concatenation strategy and L2 normalization was applied to the SSD framework to combine the intermediate inception layers to further generate new pyramid feature maps. The effectiveness of the weighted feature fusion was confirmed on three annotated testing datasets: WCE, CVC-ClinicDB, and ETIS-Larib. The Hyb-SSDNet framework adequately integrates low- and mid-level details, and high-level semantic information using a novel top-down feature fusion module. It also adopts one horizontal connection to reduce the number of repetitive computations, which shortens the detection time. In the future, a filtration strategy will be investigated to regularize the intermediate layer and reduce the false negative rate. Although the proposed model achieves satisfactory detection precision, the presence of the circumstances (e.g., debris, feces, and other factors) certainly influence the results as they cannot be completely omitted. This shortcoming should be examined through subsequent work, such as how to improve the performance of the model while maintaining a higher detection speed. 

## Figures and Tables

**Table 1:** Effects of different design factors of the mid-fusion block on the Hyb-SSDNet performances. The mAP is measured on the WCE, CVC-ClinicDB, and ETIS-Larib test sets.

Training Data	Testing Data	Mid-Fusion Block	Weighted Feature Fusion	Normalization	Feature Fusion	mAP (%)
		Inception-A (×2)	×	×	Concatenation	89.85
		Inception-A(×2)	✓	×	Summation	88.69
		Stem & Inception-A(×1)	×	B.Norm	Concatenation	88.42
		Stem & Inception-A(×1)	✓	×	Summation	89.28
WCE	WCE	Inception-A(×2)	✓	L2.Norm	Average	90.11
		Inception-A(×2)	✓	B.Norm	Average	89.96
		Inception-A(×2)	✓	L2.Norm	Concatenation	**93.29**
		Stem & Inception-A(×2)	×	B.Norm	Concatenation	89.75
		Stem & Inception-A(×2)	✓	×	Summation	91.42
		Stem & Inception-A(×2)	✓	L2.Norm	Average	92.53
		Inception-A (×2)	×	×	Concatenation	88.25
		Inception-A(×2)	✓	×	Summation	87.37
		Stem & Inception-A(×1)	×	B.Norm	Concatenation	88.21
		Stem & Inception-A(×1)	✓	×	Summation	88.98
CVC-ClinicDB		Inception-A(×2)	✓	L2.Norm	Average	89.74
&	CVC-ClinicDB	Inception-A(×2)	✓	B.Norm	Average	88.65
ETIS-Larib		Inception-A(×2)	✓	L2.Norm	Concatenation	**91.93**
		Stem & Inception-A(×2)	×	B.Norm	Concatenation	88.87
		Stem & Inception-A(×2)	✓	×	Summation	89.68
		Stem & Inception-A(×2)	✓	L2.Norm	Average	90.14
		Inception-A (×2)	×	×	Concatenation	88.49
		Inception-A(x2)	✓	×	Summation	88.03
		Stem & Inception-A(×1)	×	B.Norm	Concatenation	87.82
		Stem & Inception-A(x1)	✓	×	Summation	89.46
CVC-ClinicDB		Inception-A(×2)	✓	L2.Norm	Average	89
&	ETIS-Larib	Inception-A(×2)	✓	B.Norm	Average	88.94
ETIS-Larib		Inception-A(×2)	✓	L2.Norm	Concatenation	**91.10**
		Stem & Inception-A(×2)	×	B.Norm	Concatenation	87.73
		Stem & Inception-A(×2)	✓	×	Summation	89.41
		Stem & Inception-A(×2)	✓	L2.Norm	Average	90.05

Where B.Norm and L2. Norm mean batch normalization and L2 normalization, respectively. Concatenation is a simple concatenation strategy. Summation refers to an element-wise addition strategy, and average is an element-wise mean strategy.

**Table 2:** Comparison with previously published methods based on SSD, WCE, CVC-ClinicDB, and ETIS-Larib test sets. Pre-train means that a pre-trained backbone was adopted to initialize the model or it was initialized from scratch. The speed (FPS) and the (mAP) performances were tested using google Colab pro+ GPU.

Training Data	Methods	Backbone	Input Size	Pre-Train	FPS	mAP@0.5(%)	
						WCE	
	SSD300	VGG16	300 × 300 × 3	✓	46	77.2	
	SSD300	ResNet-101	300 × 300 × 3	✓	47.3	81.65	
	SSD500	VGG16	300 × 300 × 3	✓	19	79.45	
	SSD500	ResNet-101	300 × 300 × 3	✓	20	84.95	
WCE	FSSD300	VGG16	300 × 300 × 3	✓	65.9	89.78	
	FSSD500	VGG16	500 × 500 × 3	✓	69.6	88.71	
	DF-SSD300 [41]	DenseNet-S-32-1	300 × 300 × 3	✓	11.6	91.24	
	L_SSD [58]	ResNet-101	224 × 224 × 3	✓	40	89.98	
	MP-FSSD [18]	VGG16	300 × 300 × 3	✓	62.57	93.4	
	Hyb-SSDNet (ours)	Inception v4	299 × 299 × 3	✓	44.5	**93.29**	
						CVC-ClinicDB	ETIS-Larib
	SSD300	VGG16	300 × 300 × 3	✓	46	74.5	74.12
	SSD300	ResNet-101	300 × 300 × 3	✓	47.3	78.85	75.73
CVC-ClinicDB	SSD500	VGG16	500 × 500 × 3	✓	19	78.38	75.45
&	SSD500	ResNet-101	500 × 500 × 3	✓	20	82.74	80.14
ETIS-Larib	FSSD300	VGG16	300 × 300 × 3	✓	65.9	87.26	86.3
	FSSD500	VGG16	500 × 500 × 3	✓	69.6	87.54	86.92
	DF-SSD300 [41]	DenseNet-S-32-1	300 × 300 × 3	✓	11.6	89.92	86.84
	L_SSD [58]	ResNet-101	224 × 224 × 3	✓	40	88.18	87.23
	MP-FSSD [18]	VGG16	300 × 300 × 3	✓	62.57	89.82	90
	Hyb-SSDNet (ours)	Inception v4	299 × 299 × 3	✓	44.5	**91.93**	**91.10**

**Table 3:** WCE or colonoscopy test detection results, or both (IOU > 0.5, batch-size 1).

Training Dataset	Methods	Testing Dataset	Backbone Network	Pre-Train	Input Size	Prec	Recall	F1 Score
WCE images	Hyb-SSDNet (ours)	WCE images	Inception v4	✓	299×299	93.29%(mAP)	89.4%(mAR)	91.5%(mAF)
ETIS-Larib+CVC-ClinicDB	Hyb-SSDNet (ours)	CVC-ClinicDB	Inception v4	✓	299×299	91.93%(mAP)	89.5%(mAR)	90.8%(mAF)
ETIS-Larib+CVC-ClinicDB	Hyb-SSDNet (ours)	ETIS-Larib	Inception v4	✓	299×299	91.10%(mAP)	87%(mAR)	89%(mAF)
WCE +CVC-ClinicDB	Souaidi et al., 2022 [18]	ETIS-Larib	VGG16	✓	300×300	90.02%(mAP)	×	×
CVC-ClinicDB + ETIS-Larib	Shin et al., 2018 [59]	ETIS-Larib	Inception ResNet	✓	768×576	92.2%	69.7%	79.4%
SUN+ PICCOLO+ CVC-ClinicDB	Ishak et al., 2021 [32]	ETIS-Larib	YOLOv3	✓	448×448	90.61%	91.04%	90.82%
CVC-ClinicDB	Liu et al., 2021 [60]	ETIS-Larib	ResNet-101	✓	384×288	77.80%	87.50%	82.40%
GIANA 2017	Wang et al., 2019 [61]	ETIS-Larib	AFP-Net(VGG16)	✓	1225×996	88.89%	80.7%	84.63%
CVC-ClinicDB	Qadir et al., 2021 [62]	ETIS-Larib	ResNet34	✓	512×512	86.54%	86.12%	86.33%
CVC-ClinicDB	Pacal and Karaboga, 2021 [63]	ETIS-Larib	CSPDarkNet53	✓	384×288	91.62%	82.55%	86.85%
CVC-ClinicDB	Wang et al., 2019 [61]	ETIS-Larib	Faster R-CNN (VGG16)	×	224×224	88.89%	80.77%	84.63%
CVC-VideoClinicDB	Krenzer et al., 2019 [64]	CVC-VideoClinicDB	YOLOv5	×	574×500	73.21%(mAP)	×	79.55%

## Data Availability

Publicly available datasets were analyzed in this study. The CVC-ClinicDB datasets are publicly available here: https://polyp.grand-challenge.org/CVCClinicDB/ (accessed on 15 August 2022). The ETIS-Larib dataset is publicly available here: https://polyp.grand-challenge.org/EtisLarib (accessed on 15 August 2022).
